# Supplementary material for: Thermographic Image of the Hoof Print in Leisure and Cross-Country Warmblood Horses: A Pilot Study
Source: Vet Sci. 2023 Jul 18;10(7):470. doi: 10.3390/vetsci10070470 (PMC10385432; doi:10.3390/vetsci10070470)
Supplement: Supplementary file 1 [file vetsci-10-00470-s001.zip › Etic and project approval.pdf]

The Bioethics Commission

Banat University of Agricultural Sciences and Veterinary Medicine „King Michael I of Romania" from Timișoara

119, Calea Aradului, 300645, Timișoara, RO ▪ tel/fax: +40 256 277140

ROMÂNIA
UNIVERSITATEA DE ȘTIINȚELE VIEȚII "REGELE MIHAI I" DIN TIMIȘOARA
RECTORAT
MINISTERUL EDUCAȚIEI

The Scientific Pro-Rectorate

No. 51 of 07.06.2021

# To whom it may concern

The Bioethics Commission from Banat University of Agricultural Sciences and Veterinary Medicine „King Michael I of Romania" from Timișoara, analyzed and approved the experimental protocols consisting in studies on horses in the period June 2021 to December 2022 for the project: **"Research for Diagnostic and Treatment of the Acropodial Pathology in Horses"** conducted *by* ***Assistant professor Zaha Cristian,*** *DVM from the Faculty of Veterinary Medicine Timisoara, Dept. of Surgery.*

The experiments assure the proper accommodation and animal handling in accordance to the Directive 2010/63/EU. Also the experimental protocol includes methods for preventing and reducing animal distress in accordance to the recommendations of European Union and Romanian legislation.

We believe that the proposed study can be carried out under the conditions described in the protocol and complies with the recent regulations on protection of animals used for experimental and other scientific purposes.

Head of
Bioethics Commission of BUASVM Timișoara
