# Supplementary material for: Thermographic Image of the Hoof Print in Leisure and Cross-Country Warmblood Horses: A Pilot Study
Source: Vet Sci. 2023 Jul 18;10(7):470. doi: 10.3390/vetsci10070470 (PMC10385432; doi:10.3390/vetsci10070470)
Supplement: Supplementary file 1 [file vetsci-10-00470-s001.zip › Table S1 - Horse information.pdf]

Number	Breed	Performance type	Age	Weight	Height	Lameness Score
1	Warmblood – Romanian Sport Horse	Leisure	12	564	162	Negative reaction to lameness examination
2	Warmblood – Romanian Sport Horse	Leisure	4	512	154	Negative reaction to lameness examination
3	Warmblood – Romanian Sport Horse	Leisure	16	550	158	Score 2
4	Warmblood – Romanian Sport Horse	Leisure	7	517	155	Negative reaction to lameness examination
5	Warmblood – Romanian Sport Horse	Leisure	7	544	161	Negative reaction to lameness examination
6	Warmblood-Lipizzaner	Leisure	9	615	165	Negative reaction to lameness examination
7	Warmblood-Oldenburg	Leisure	11	587	164	Score 2
8	Warmblood-Lipizzaner	Leisure	8	540	160	Negative reaction to lameness examination
9	Warmblood-Lipizzaner	Leisure	10	532	158	Negative reaction to lameness examination
10	Warmblood – Romanian Sport Horse	Leisure	11	514	156	Score 1
11	Warmblood-Oldenburg	Leisure	8	525	168	Negative reaction to lameness examination
12	Warmblood-Lipizzaner	Leisure	8	514	162	Negative reaction to lameness examination
13	Warmblood – Romanian Sport Horse	Leisure	6	517	159	Score 2
14	Warmblood-Oldenburg	Leisure	11	572	160	Negative reaction to lameness examination
15	Warmblood – Romanian Sport Horse	Leisure	12	557	160	Score 1
16	Warmblood-Oldenburg	Leisure	8	578	164	Negative reaction to lameness examination
17	Warmblood-Lipizzaner	Leisure	7	580	158	Negative reaction to lameness examination
18	Warmblood-Lipizzaner	Leisure	7	584	161	Score 2
19	Warmblood-Lipizzaner	Leisure	8	567	154	Score 2
20	Warmblood – Romanian Sport Horse	Leisure	6	532	155	Negative reaction to lameness examination
21	Warmblood – Romanian Sport Horse	Leisure	7	542	158	Negative reaction to lameness examination
22	Warmblood-Oldenburg	Leisure	7	595	160	Negative reaction to lameness examination
23	Warmblood-Oldenburg	Leisure	5	570	164	Negative reaction to lameness examination

24	Warmblood-Oldenburg	Leisure	6	547	158	Score 1
25	Warmblood – Romanian Sport Horse	Leisure	4	526	148	Negative reaction to lameness examination
26	Warmblood – Romanian Sport Horse	Leisure	8	515	162	Negative reaction to lameness examination
27	Warmblood – Romanian Sport Horse	Leisure	7	565	164	Negative reaction to lameness examination
28	Warmblood – Romanian Sport Horse	Leisure	9	615	162	Score 2
29	Warmblood – Romanian Sport Horse	Leisure	11	528	156	Score 3
30	Warmblood-Lipizzaner	Leisure	6	576	162	Negative reaction to lameness examination
31	Warmblood-Lipizzaner	Leisure	7	560	152	Score 1
32	Warmblood-Lipizzaner	Leisure	8	564	164	Negative reaction to lameness examination
33	Warmblood – Romanian Sport Horse	Leisure	6	536	160	Negative reaction to lameness examination
34	Warmblood – Romanian Sport Horse	Leisure	14	517	156	Negative reaction to lameness examination
35	Warmblood – Romanian Sport Horse	Leisure	14	540	159	Score 1
36	Warmblood – Romanian Sport Horse	Leisure	11	545	162	Score 2
37	Warmblood – Romanian Sport Horse	Leisure	5	535	164	Negative reaction to lameness examination
38	Warmblood – Romanian Sport Horse	Leisure	8	515	157	Negative reaction to lameness examination
39	Warmblood – Romanian Sport Horse	Leisure	4	524	158	Score 1
40	Warmblood-Lipizzaner	Leisure	5	562	162	Negative reaction to lameness examination
41	Warmblood-Lipizzaner	Leisure	6	575	158	Negative reaction to lameness examination
42	Warmblood-Oldenburg	Leisure	7	565	167	Score 1
43	Warmblood – Romanian Sport Horse	Leisure	8	535	159	Negative reaction to lameness examination
44	Warmblood-Oldenburg	Leisure	6	541	161	Negative reaction to lameness examination
45	Warmblood-Lipizzaner	Leisure	7	575	164	Negative reaction to lameness examination

46	Warmblood-Lipizzaner	Leisure	8	556	162	Score 2
47	Warmblood – Romanian Sport Horse	Leisure	7	535	164	Negative reaction to lameness examination
48	Warmblood – Romanian Sport Horse	Leisure	6	526	162	Negative reaction to lameness examination
49	Warmblood-Lipizzaner	Leisure	7	521	159	Negative reaction to lameness examination
50	Warmblood-Lipizzaner	Leisure	7	548	159	Negative reaction to lameness examination
51	Warmblood-Lipizzaner	Leisure	6	546	152	Negative reaction to lameness examination
52	Warmblood-Oldenburg	Leisure	5	568	158	Negative reaction to lameness examination
53	Warmblood – Romanian Sport Horse	Cross-country	5	535	164	Score 2
54	Warmblood – Romanian Sport Horse	Cross-country	6	542	162	Negative reaction to lameness examination
55	Warmblood-Lipizzaner	Cross-country	4	538	164	Negative reaction to lameness examination
56	Warmblood-Lipizzaner	Cross-country	5	564	160	Score 1
57	Warmblood-Lipizzaner	Cross-country	7	524	158	Negative reaction to lameness examination
58	Warmblood – Romanian Sport Horse	Cross-country	6	534	163	Negative reaction to lameness examination
59	Warmblood-Oldenburg	Cross-country	6	545	160	Negative reaction to lameness examination
60	Warmblood-Oldenburg	Cross-country	8	564	168	Score 2
61	Warmblood – Romanian Sport Horse	Cross-country	7	520	158	Negative reaction to lameness examination
62	Warmblood – Romanian Sport Horse	Cross-country	8	529	162	Score 2
63	Warmblood-Lipizzaner	Cross-country	9	542	161	Negative reaction to lameness examination
64	Warmblood-Lipizzaner	Cross-country	9	545	158	Negative reaction to lameness examination
65	Warmblood-Lipizzaner	Cross-country	12	562	154	Score 2
66	Warmblood-Oldenburg	Cross-country	11	550	168	Score 1
67	Warmblood-Oldenburg	Cross-country	6	524	162	Negative reaction to lameness examination
68	Warmblood-Lipizzaner	Cross-country	8	541	158	Negative reaction to lameness examination
69	Warmblood-Lipizzaner	Cross-country	5	563	165	Negative reaction to lameness examination
70	Warmblood – Romanian Sport Horse	Cross-country	9	517	158	Negative reaction to lameness examination

71	Warmblood – Romanian Sport Horse	Cross-country	7	528	162	Negative reaction to lameness examination
72	Warmblood – Romanian Sport Horse	Cross-country	8	532	158	Score 1
73	Warmblood-Oldenburg	Cross-country	8	534	162	Negative reaction to lameness examination
74	Warmblood-Oldenburg	Cross-country	9	561	162	Negative reaction to lameness examination
75	Warmblood-Oldenburg	Cross-country	4	526	164	Negative reaction to lameness examination
76	Warmblood-Oldenburg	Cross-country	6	542	167	Negative reaction to lameness examination
77	Warmblood – Romanian Sport Horse	Cross-country	7	537	157	Negative reaction to lameness examination
78	Warmblood – Romanian Sport Horse	Cross-country	8	546	161	Negative reaction to lameness examination
79	Warmblood – Romanian Sport Horse	Cross-country	10	536	164	Score 2
80	Warmblood-Oldenburg	Cross-country	9	532	162	Negative reaction to lameness examination
81	Warmblood – Romanian Sport Horse	Cross-country	5	518	158	Negative reaction to lameness examination
82	Warmblood-Lipizzaner	Cross-country	8	536	162	Negative reaction to lameness examination
83	Warmblood-Lipizzaner	Cross-country	6	542	160	Score 2
84	Warmblood-Lipizzaner	Cross-country	7	545	162	Negative reaction to lameness examination
85	Warmblood-Oldenburg	Cross-country	7	536	166	Score 1
86	Warmblood-Oldenburg	Cross-country	8	545	162	Negative reaction to lameness examination
87	Warmblood-Oldenburg	Cross-country	9	552	167	Score 1
