# Supplementary figures and images for: Thermographic Image of the Hoof Print in Leisure and Cross-Country Warmblood Horses: A Pilot Study
Source: Vet Sci. 2023 Jul 18;10(7):470. doi: 10.3390/vetsci10070470 (PMC10385432; doi:10.3390/vetsci10070470)

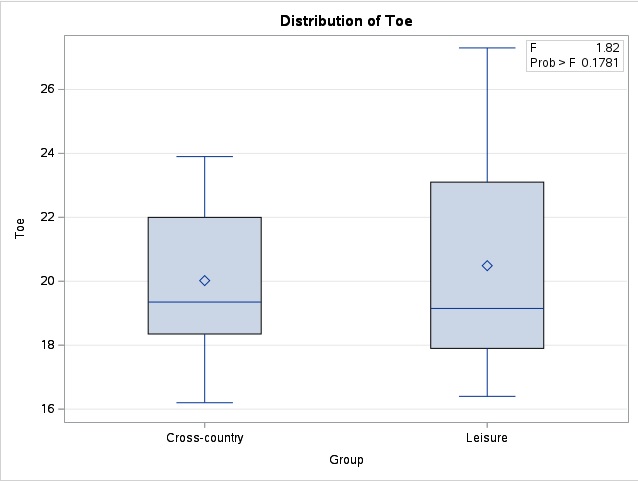

Supplement: Supplementary file 1 [file vetsci-10-00470-s001.zip › Figure S1 - Leisure vs. Cross Country - Toe Results One-Way ANOVA.jpg]

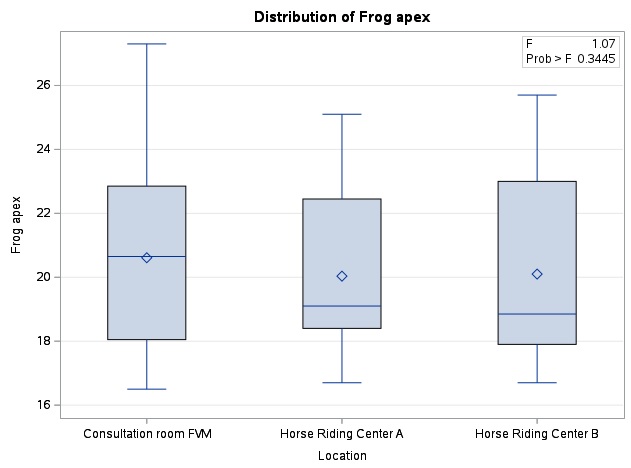

Supplement: Supplementary file 1 [file vetsci-10-00470-s001.zip › Figure S10 - Comparison between location - Frog Apex Results One-Way ANOVA.jpg]

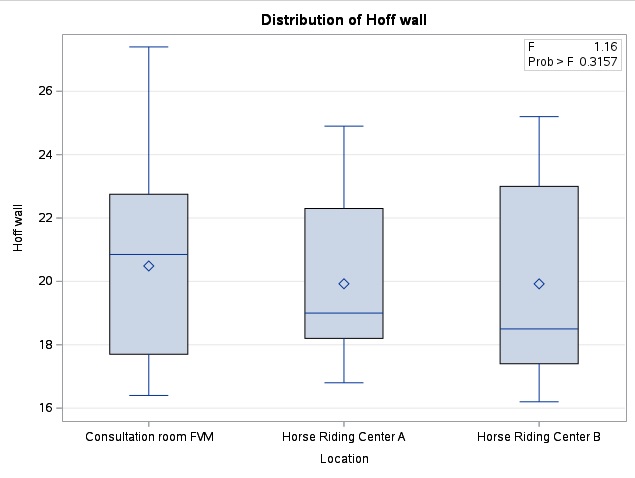

Supplement: Supplementary file 1 [file vetsci-10-00470-s001.zip › Figure S11 - Comparison between location - Hoof wall Results One-Way ANOVA.jpg]

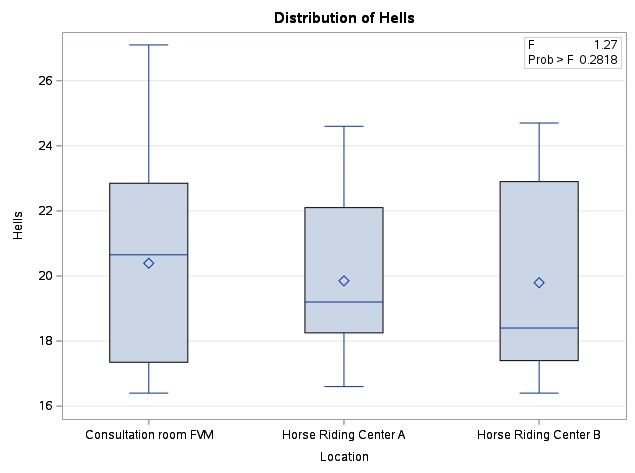

Supplement: Supplementary file 1 [file vetsci-10-00470-s001.zip › Figure S12 - Comparison between location - Heels - Results One-Way ANOVA.jpg]

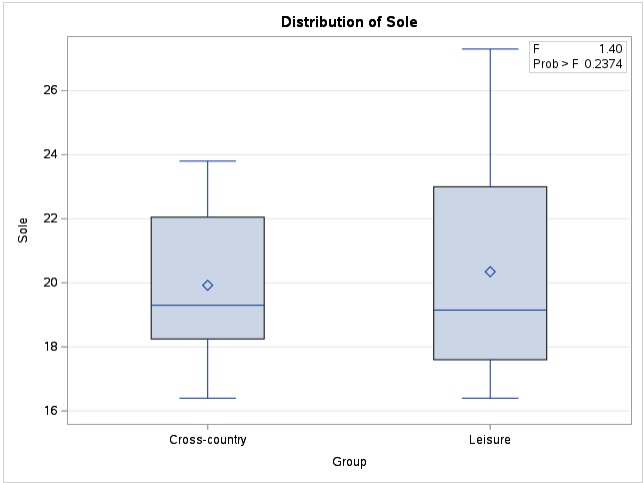

Supplement: Supplementary file 1 [file vetsci-10-00470-s001.zip › Figure S2 - Leisure vs. Cross Country - Sole Results One-Way ANOVA.jpg]

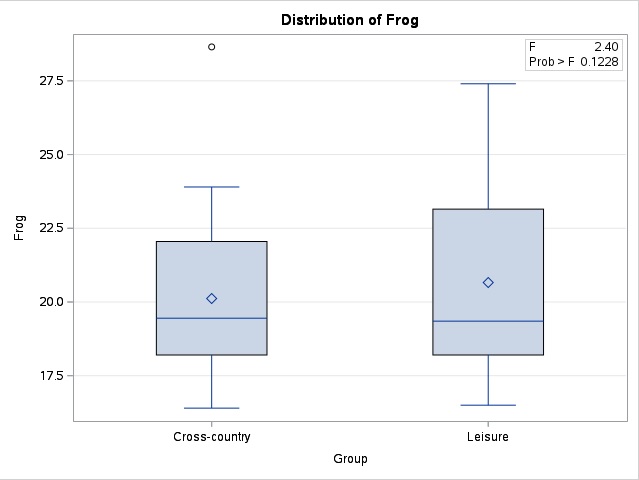

Supplement: Supplementary file 1 [file vetsci-10-00470-s001.zip › Figure S3 - Leisure vs. Cross Country - Frog Results One-Way ANOVA.jpg]

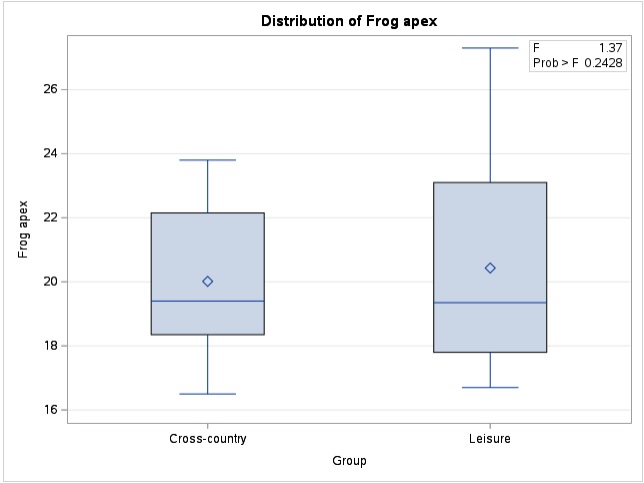

Supplement: Supplementary file 1 [file vetsci-10-00470-s001.zip › Figure S4 - Leisure vs. Cross Country - Frog apex -Results One-Way ANOVA.jpg]

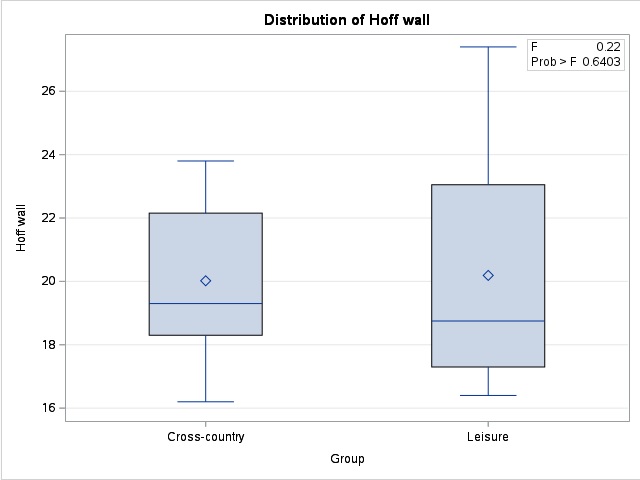

Supplement: Supplementary file 1 [file vetsci-10-00470-s001.zip › Figure S5 - Leisure vs. Cross Country - Hoof Wall Results One-Way ANOVA.jpg]

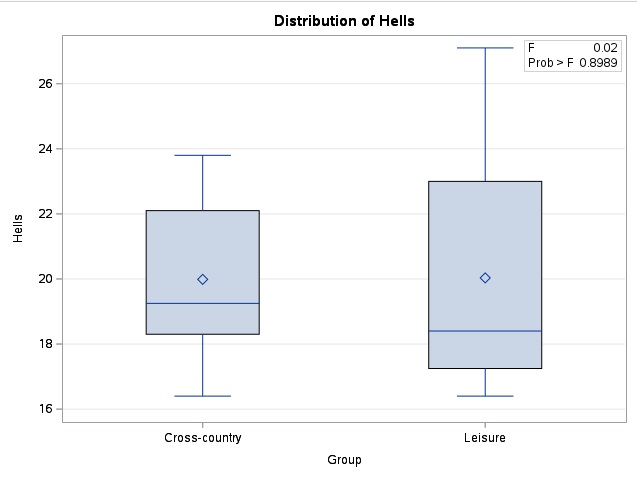

Supplement: Supplementary file 1 [file vetsci-10-00470-s001.zip › Figure S6 - Leisure vs. Cross Country - Heels Results One-Way ANOVA.jpg]

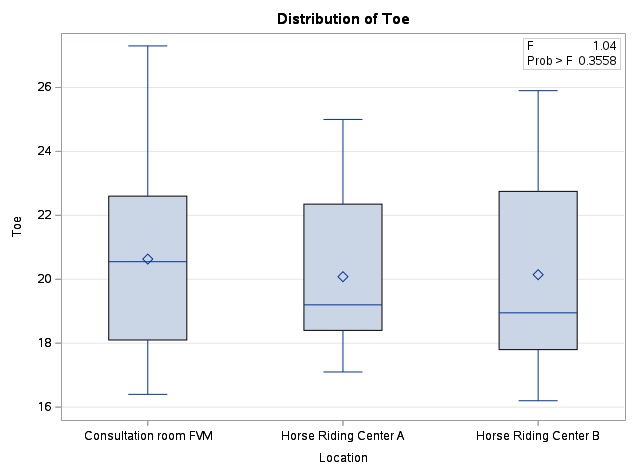

Supplement: Supplementary file 1 [file vetsci-10-00470-s001.zip › Figure S7 - Comparison between location - Toe Results One-Way ANOVA.jpg]

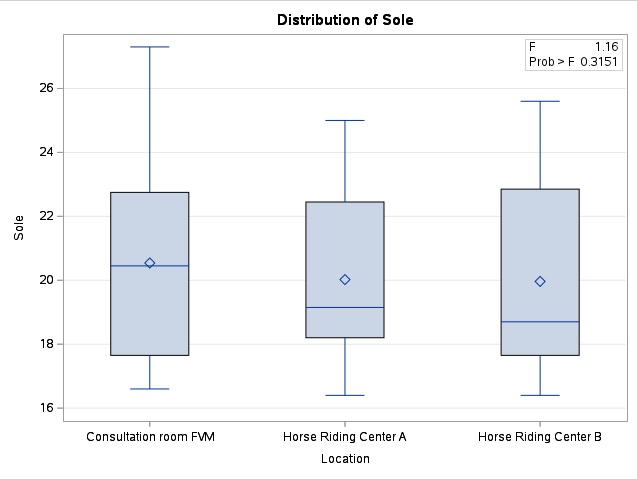

Supplement: Supplementary file 1 [file vetsci-10-00470-s001.zip › Figure S8 - Comparison between location - Sole - Results One-Way ANOVA.jpg]

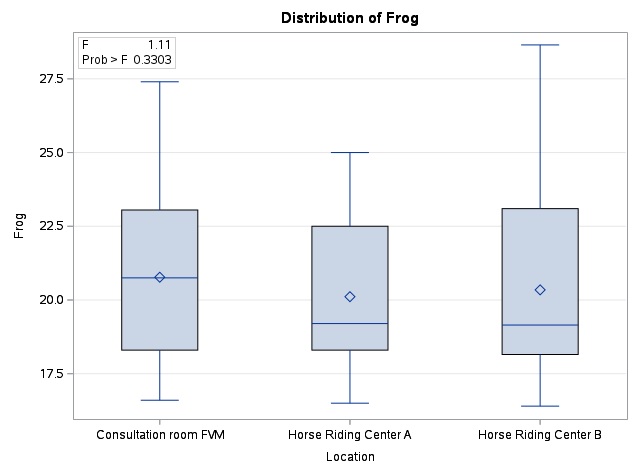

Supplement: Supplementary file 1 [file vetsci-10-00470-s001.zip › Figure S9 - Comparison between location - Frog - Results One-Way ANOVA.jpg]
